# Subretinal Transplant of Human Amniotic Membrane in Advanced Age-Related Macular Degeneration

**DOI:** 10.3390/life12121998

**Published:** 2022-11-30

**Authors:** Tomaso Caporossi, Alessandra Scampoli, Antonio Baldascino, Gloria Gambini, Bianca Pacini, Lorenzo Governatori, Daniela Bacherini, Matteo Mario Carlà, Emanuele Crincoli, Clara Rizzo, Raphael Kilian, Stanislao Rizzo

**Affiliations:** 1 Ophthalmology Unit, “Fondazione Policlinico Universitario A. Gemelli IRCCS”, 00168 Rome, Italy; 2 Ophthalmology Unit, Catholic University “Sacro Cuore”, 00168 Rome, Italy; 3 Department of NEUROFARBA, Ophthalmology, University of Florence, Careggi, 50139 Florence, Italy; 4 Ophthalmology Unit, Department of Surgical, Medical, Molecular and Critical Area Pathology, University of Pisa, 56122 Pisa, Italy; 5 Ophthalmology Unit, University of Verona, 37129 Verona, Italy; 6 Consiglio Nazionale delle Ricerche, Istituto di Neuroscienze, 56124 Pisa, Italy

**Keywords:** age-related macular degeneration, macular neovascularization, stem cells, regenerative ophthalmology, human amniotic membrane, OCT-angiography, vitrectomy

## Abstract

Macular neovascularization (MNV) and geographic atrophy can complicate age-related macular degeneration (AMD) and lead to severe visual acuity reduction. Despite the medical treatments available, with a defect in the retinal pigmented epithelium (RPE) there is no possibility of restoring acceptable visual acuity. We evaluated postoperative outcomes in patients affected by advanced AMD who underwent subretinal implant of the human amniotic membrane (hAM) as a source of pluripotent stem cells. This retrospective, consecutive, non-randomized interventional study included 23 eyes of 21 patients affected by AMD complicated by MNV, and five eyes of five patients affected by geographic atrophy. All eyes underwent a pars plana vitrectomy, neovascular membrane removal for the MNV group, a subretinal implant of hAM, and gas tamponade, and were followed for 12 months. The primary study outcome was visual acuity improvement. Secondary outcomes were postoperative complications, OCT-angiography parameters correlated with best-corrected visual acuity (BCVA) and MNV recurrence. The mean preoperative BCVA was 1.9 logMAR, and the mean final BCVA value was 1.2 logMAR. In the MNV group, the mean BCVA improved from 1.84 logMAR to 1.26 logMAR, and from 1.84 logMAR to 1.32 logMAR in the geographic atrophy group. No MNV recurrence was evident in 12 months of follow-up. An OCT-angiography scan was used to evaluate the retinal vascularization in the treated eye, which showed a high correlation between BCVA and deep vascular density. This study demonstrates the hAM potential and safety in promoting a partial restoration of retinal function together with an increase in visual acuity.

## 1. Introduction

Age-related macular degeneration (AMD) is the main factor contributing to irreversible blindness in the population 65 years of age and older [1]. Macular neovascularization (MNV) can exacerbate AMD and cause a significant loss of visual acuity. There are currently a variety of MNV treatments available, including anti-VEGF intravitreal injections [2,3]; although medical treatments are not enough in the case of damaged retinal pigmented epithelium (RPE). In 2020, Rizzo et al. proposed a new surgical approach involving the use of the human amniotic membrane (hAM) as a support to restore damaged photoreceptors in AMD on a sample of 11 patients, which showed encouraging results [4].

The hAM Is the innermost translucid layer of the placenta and is 0.02–0.05 mm thick. It is defined by a basement membrane made of type IV and VII collagen, hyaluronic acid, fibronectin, and laminin, an epithelium made of cuboidal cells, plenty of microvilli with active metabolism, and an avascular stroma [5]. Human amniotic epithelial cells (hAECs) are foetal in principle, so they exhibit a high level of pluripotency. hAECs are an easily accessible, non-invasive, and cost effective cell source for clinical use that can be obtained from the epithelial layer of the hAM. The low surface expression of human leukocyte antigens (HLA)-A, HLA-B, HLA-C, and HLA-DR, which are associated with post-transplant rejection, accounts for the hAECs’ immunological inertness. They also have a high surface expression of HLA-G, which is a non-classical antigen that can repress the immune response [6]. Furthermore, hAECs do not form neoplasia in vivo as they lack the telomerase enzyme and do not differentiate into fibroblasts [6]. These properties make it possible to safely transplant hAECs into people without worrying about an obvious immunological reaction.

Numerous studies demonstrate that a vast spectrum of growth factors, including the transforming growth factors (TGF-1, TGF-2, and TGF-3), the basic fibroblast growth factor, and the epithelial growth factor, can be produced by the amniotic membrane [7,8]. When surgically implanted, the hAM represents a reservoir of neurotrophic factors, such as nerve growth factor (NGF), brain-derived neurotrophic factor (BDNF), neurotrophin 3 (NT-3), glial cell-derived neurotrophic factor (GDNF), and ciliary neurotrophic factor (CNTF) [9]. Various surgical subspecialties have employed the amniotic membrane. hAM has been applied to cover retinal diseases, sterile melts, and corneal ulcers for ophthalmic surgery [10,11,12,13,14,15,16,17]. The hAM has demonstrated impressive repairing abilities under these circumstances, with hypothesized, but still to be demonstrated, regenerating features. It also exhibits anti-inflammatory, anti-angiogenic, and anti-microbial properties [18,19].

In this work, we wanted to demonstrate that advanced AMD-related retinal and RPE degeneration can be treated with transplanted hAM plugs that can encourage photoreceptor regeneration. We previously began to demonstrate this in a sample of 11 eyes affected by advanced AMD with MNV and geographic atrophy [4]. However, the present study is based on a larger cohort of eyes.

## 2. Materials and Methods

### 2.1. Patients

This paper reports a retrospective, interventional, non-randomized consecutive case-series study conducted at the Fondazione Policlinico Universitario A. Gemelli-IRCCS, Rome and Careggi Hospital, Florence University from 12 January 2019 and 21 March 2021. Each patient signed an informed consent form to participate in this study, which was adherent to the Declaration of Helsinki. We included 28 eyes of 26 patients who suffered from advanced AMD, complicated with MNV in 23 eyes, and underwent vitrectomy with hAM implantation.

Inclusion criteria were the presence of a fibrous or fibro-haemorrhagic subretinal MNV evaluated by optical coherence tomography (OCT), showing no structural changes over a 6-month period free of intravitreal injections (IVTs), or the presence of atrophic maculopathy. Those patients were divided into two groups (MNV-group and GA-group).

The following exclusion criteria were considered: age less than 18; a history of intraocular inflammation or retinal abnormalities; and a history of retinal detachment or macular pathologies different from AMD (such as epiretinal membranes, macular holes, and vitreo-macular traction).

### 2.2. Study Exams

An eye history and examination, including refraction and an evaluation of the best-corrected visual acuity (BCVA) using Snellen charts, were obtained prior to surgery and at the 1-, 3-, 6-, and 12-month follow-ups. For statistical analysis, the Snellen values were transformed into the logarithm of the minimum angle of resolution (logMAR) values. In cases of low BCVA, we assigned a logMAR value according to Holladay [20]. The intraocular pressure was measured using Goldmann applanation tonometry (IOP).

At baseline, along with standard dilated fundus ophthalmic examination, spectral domain OCT (AngioVue Optovue, Fremont, CA, USA) and OCT-angiography (OCT-A) (AngioVue Optovue, Fremont, CA, USA) were performed after surgery using 3 mm × 3 mm and 6 mm × 6 mm section scanning of the foveal area. Based on the ETDRS grid sectors, the superficial capillary plexus (SCP) and deep capillary plexus (DCP) were analyzed, and results were reported as the proportion of area occupied by vessels in the total, foveal, and parafoveal areas. Optovue was utilized to evaluate the angiography and calculate the foveal avascular zone (FAZ) area. Moreover, each afflicted eye underwent a microperimetry examination (MP-3 Nidek Co., Ltd., Maehama, Japan). Microperimetry stimulus parameters settings were Goldmann III, 200 ms duration, and a 4–2 staircase. The MP-3 has a background illumination of 31.4 asb (10 cd/m^2^). A pre-set standard grid, which covers the macular 10°, was used, made of three concentric rings located at a distance of 1°, 6°, and 10° from a central stimulus, for a total of 40 stimuli. The evaluations were completed in a dark room with the opposite eye covered. In the event of considerable eye movements, which happened often in individuals with AMD owing to poor fixation, the test was immediately halted. After successfully realigning the subject’s eye, the test could continue.

All those exams were repeated at the 12-month follow-up. All OCT and OCT-A images were captured with the in-built follow-up option of the AngioVue Optovue and repeated several times to obtain the best possible quality. Moreover, in order to avoid possible misalignments, all scans were manually double-checked after data.

Finally, an adaptive optics scan (Rtx1, Imagine Eyes, France) was performed 12 months after surgery, to check the possible presence of photoreceptors over and around the hAM patch.

The main outcome of the study was the visual acuity improvement between before the operation and 12 months after using BCVA. The secondary outcomes were postoperative complications, OCT-angiography analysis and how it correlated with BCVA, microperimetry variations analysis, and eventual MNV recurrence report.

### 2.3. Statistical Analysis

The statistical analysis was conducted using STATA software version 15.1 (StataCorp, College Station, TX, USA). Our sample’s normality was determined using the Shapiro–Wilk test, and *p* > 0.05 was utilized to confirm the null hypothesis. We conducted an Analysis of Variance (ANOVA) and employed the Dunnett’s multiple comparison test to evaluate the differences among retinal parameters in different follow-ups. Furthermore, a Tukey test, computing confidence intervals, was used to compare the difference between each pair of means. For contingency analysis, chi-square and Fisher’s exact tests were utilized. Quantitative values were expressed as mean ± SD and a *p*-value < 0.05 was considered statistically significant. A designated confidence interval (CI) of 95% was used. The significance threshold was established at *p* = 0.05.

### 2.4. Surgical Technique

Under retrobulbar anaesthesia, the patients underwent a standard 3-port, 23-gauge pars plana vitrectomy (PPV) (Constellation; Alcon Laboratories, Fort Worth, TX, USA), and a 25-gauge chandelier endoilluminator was inserted to facilitate bimanual manoeuvres. Following proper vitreal base shaving, with a maximum injection pressure of 30 PSI, a 41-gauge needle was attached to a 10-cc syringe containing balancing 1 saline solution (BSS) and coupled to the viscous fluids injecting circuit of the Constellation^®^ system. Fluid was injected under the retina to create a retinal detachment, and many air-fluid exchanges were done to displace the subretinal fluid and achieve a sufficient retinal detachment.

A posterior retinectomy was performed outside the supero-temporal retinal vascular arcade using vitreous scissors after accurate diathermy. A curved laser probe was inserted under the retina trough the retinectomy to gently detach the retina form the membrane adherences. In the MNV group, the neovascular tissue was extracted from the retina with vitreous forceps and removed using a vitrectomy probe. In the GA group, retinal detachment was restricted to the posterior pole, and the retina was gently detached from the underlying atrophic scar using a smooth cannula or spatula introduced in the retinectomy.

The hAM was provided by the Eye Bank of Lucca and the Eye Bank of Rome, Italy. A hAM disk with 5 mm diameter was prepared with a cutaneous punch (Disposable Biopsy Punch, Kai Medical, Solingen, Germany) and inserted through a 23-gauge trocar. Using two vitreous forceps (Greishaber, Alcon Laboratories, Fort Worth, TX, USA), the hAM plug was transplanted in the macular area with the chorion layer facing the RPE trough the retinectomy. Once the chorion layer had adhered to the RPE, the hAM patch remained stretched out. The retina was reattached over the hAM patch using Perfluoro-N-Octane (DORC International, Zuidlan, The Netherlands), and laser retinopexy was performed at the edge of the retinectomy area. In all cases, 20% sulphur-hexafluoride (SF_6_) (Fluoron GmbH, Germany) was used as an endotamponade (see Supplementary Materials Video S1). The patients were asked to maintain a face-down position for the first week.

In all phakic patients, a combined phacoemulsification was performed just before vitrectomy.

## 3. Discussion

Embryonic stem cells (ESCs) [21] and induced pluripotent stem cells (iPSCs) [22] are a recent and intriguing field of study in AMD treatment. According to the literature, the transplantation of allogeneic RPE cells into the subretinal region causes an immune reaction that impairs the cells’ ability to survive and, in some circumstances, results in a fibrotic reaction that worsens the macular state [23]. In contrast, the transplantation of hAM has demonstrated good safety and a non-immunogenic profile [6].

Recently, human RPE cells were cultivated in vitro on a hAM sheet, demonstrating the viability of this material as a substrate for the growth and proliferation of the RPE. RPE cells, while maintaining epithelial characteristics, generated a highly organized monolayer [24] and produced a number of growth factors required to keep retinal homeostasis in place [25].

After surgically removing the RPE and mechanically tearing the Bruch’s membrane, Kiilgaard et al. positioned hAM in the subretinal area of a pig. They demonstrated that there was barely any inflammation and that the hAM was well tolerated. The host’s RPE was in contact with a monolayer of pigmented cells that covered it, considerably reducing choroidal neovascularization [26]. In a traumatic globe rupture in 2017, Zhu et al. investigated a hAM plug for the treatment of suprachoroidal silicone oil migration associated to a choroidal hole. Three months following the procedure, they showed how the membrane served as a mechanical scaffold, stimulating the creation of choroid vascular tissue and collagen [27].

Recently, it has been proposed that the hAM could be used to repair refractory macular holes or peripheral retinal tears that cause retinal detachments (RD) [12]. In cases of macular holes, optical coherence tomography (OCT) images show the regeneration of the retinal layers with a cellular migration above the amniotic membrane. This suggests there is promise in using hAM in terms of partial regenerative capacity for retinal layers.

Based on the pluripotency of the hAM, in 2020, we started to test an innovative approach involving the use of a hAM patch positioned under the foveal area as a support to restore damaged photoreceptors in MNV and geographic atrophy in a sample of 11 patients [4]. Driven by the encouraging results, we expanded the patient sample to 28 eyes and focused on the ability of the hAM to induce a possible regenerative process in patients affected by AMD. The Bruch’s membrane that is destroyed throughout the AMD process might theoretically be restored by our approach, which involves placing a hAM basal membrane on the choroidal side. Additionally, the preservation of the same macular retinal pigmented epithelium may aid in the regeneration of the retinal layers over the hAM.

Using human embryonic stem cells, da Cruz and associates bioengineered a sheet of retinal pigmented epithelial cells (h-ESC). A single layer of RPE cells placed on a synthetic basement membrane were created by differentiating the cells. Then, two patients with advanced AMD received this monolayer implant. They produced positive anatomical results, but rather modest functional ones. Additionally, the patients had to take perioperative prednisone (systemic immunosuppressive medication) for at least a year. Instead, we avoided using any steroid medication in our series.

The Bruch’s membrane that is lost throughout the CNV in the AMD process may be restored by our approach, which involves inserting the hAM basal membrane on the choroidal side. Additionally, in order to drive the RPE cell monolayer toward the neuroepithelium, da Cruz and associates installed the artificial basal membrane on the choroidal side [21].

Using a sub-retinal implant of a polarized monolayer of hESC-RPE on a nonbiodegradable, synthetic parylene substrate, Kashani et al. have treated four patients of advanced AMD. In order to verify the existence of the hESC-RPE implant and the ELM regeneration, they reported an OCT study. In two out of four patients, a fixation test revealed an improvement from an unstable fixation to a stable fixation following the implantation. Three individuals improved their VA to 1.2 logMAR (20/300), the mean preoperative BCVA recorded was 1.3 logMAR (20/400), and one patient did not have any improvement in vision as a result of a postoperative subretinal hemorrhage [28].

Compared to the challenging technique of RPE-choroid graft translocation proposed by Van Zeeburg, Parolini, and Van Romunde [29,30,31], and since there is no need to execute macular translocation maneuvers, harvest retinal pigmented epithelium or choriocapillaris free flaps, or implant hAM plugs, the procedure is simpler.

In 40% of patients, Parolini reported satisfactory anatomical and functional outcomes with ultimate visual acuity of 20/200. Their method had a number of issues, including macular diseases and retinal detachment.

Van Romunde claimed similar outcomes attained in 81 people utilizing a comparable method. The final average BCVA was 20/160; 89% of the hemorrhagic patients and 46% of the atrophic patients saw an improvement in BCVA [31].

Our surgical procedure is carried out using less retinal tissue manipulation, which may result in a lower PVR stimulus. We observed BCVA improvement in 23 of the 28 eyes (82.14%) with a mean final BCVA of 20/300. Five eyes (17.8%) had no changes in BCVA, and none showed worsening BCVA. These results suggest that the hAM triggered a possible regenerative process that improved BCVA.

When analyzing OCT-angiography to examine the retinal vascularization following subretinal hAM implantation, we saw an increase in the deep capillary plexus’ vascular density, which seemed to be connected to BCVA. This outcome supported the conclusions of several other investigations, including those of Wakabayashi et al. They confirmed that the DCP density is the main variable linked with visual acuity in vascular macular disorders. Additionally, these disorders during the early stages, largely damage the plexus [32].

We have shown that there is a strong association between the BCVA and DVD values obtained with OCT-A in the macular region. However, the measured SVD values have not shown any correlation with the final BCVA. When a steady central fixation was achieved, microperimetry showed that the retina responded to stimuli in the regions implicated in the recovery process over the hAM patch. The results of the microperimetry were verified by the AO pictures, which showed a repopulation or migration of photoreceptors beginning at the hAM margins.

This study has several limitations, due its retrospective design, the small sample size, and the absence of a control group, resulting in non-comparative findings. Moreover, intermediate follow-ups lack instrumental analyses, such as microperimetry and OCT-A. However, this research could be a starting point in optimizing severe AMD management. Natural visual prognosis of severe AMD indeed determines an anticipated VA loss of three lines within three years [33]. According to the Submacular Surgery Trial, an operation that completely removed the neovascular complex, blood, and fibrovascular scar tissue did not raise the likelihood that the patient’s VA would improve. Instead, it just decreased the likelihood of suffering a significant VA loss, which remained consistent throughout the observations [34]. Our study showed VA improvement in all patients affected by advanced MNV, which was most likely caused by both the loss of blood or fibrovascular tissue, as well as the support of the AM plug and its neurotrophic properties.

## 4. Results

A total of 28 eyes of 26 patients were enrolled. A hemorrhagic MNV caused 23 cases of fibro-hemorrhagic subretinal membrane and 5 cases of atrophic maculopathy. In the MNV group, two patients had surgery on both eyes. The mean age was 79.1 ± 5.8 years (range, 68–88 years), 13 were men and 13 women. No cases of intraoperative complications were reported.

The mean preoperative best corrected visual acuity (BCVA) was 1.88 logMAR (20/2000 Snellen) ranging from 20/20,000 to 20/200 (3–1 logMAR). The average 1-month follow-up BCVA was 1.61 logMAR (20/1000 Snellen), ranging from 3 to 0.9 logMAR (20/20,000–20/200 Snellen). The average 3-month follow-up BCVA was 1.4 logMAR (20/500 Snellen), ranging from 3 to 0.7 logMAR (20/20,000–20/100 Snellen). The mean 6-month follow-up BCVA was 1.32 logMAR (20/400 Snellen), ranging from 20/20,000 to 20/100 (2–0.7 logMAR). The final mean BCVA was 1.23 logMAR (20/400 Snellen), ranging from 20/20,000 to 20/63 (3–0.5 logMAR) (Table 1 and Table 2).

Four patients in the MNV group and one patient in the atrophic group experienced no BCVA gain during the course of the 12-month follow-up. In the follow-up, OCT revealed that the hAM patch was not positioned sub-macularly. Neither functional improvement utilizing microperimetry nor repair of the external limiting membrane (ELM) were observed in these cases.

Four patients developed an epiretinal membrane (ERM) formation, but none of them required surgical treatment.

The difference between pre- and postoperative BCVA was statistically significant in both the hemorrhagic and the atrophic group. Moreover, no MNV reactivation and no additional anti-VEGF injections were noted after a 12-month period of follow-up.

Finally, a subgroup analysis of patients undergoing combined surgery (phacoemulsification and vitrectomy) was conducted, but no statistical differences were found between this group and the vitrectomy-alone group in terms of visual gain (*p* = 0.48).

### 4.1. MNV-Group

There were 23 eyes of 21 patients that met the inclusion criteria. Before surgery, all patients had a history of receiving several anti-VEGF injections. There were 11 women, 10 males, 15 right eyes, 8 left eyes, and 9 phakic eyes (39.1%), and the mean age was 78.76 ± 6.13 years (range 68–88 years).

The mean preoperative BCVA was 1.9 ± 0.4 logMAR (20/2000 Snellen) with a range of 3 to 1 logMAR (20/20,000–20/200 Snellen). The 1-month BCVA was 1.6 ± 0.5 (range 3–0.9), the 3-month BCVA was 1.4 ± 0.5 (range 3–0.7), the 6-month BCVA was 1.3 ± 0.5 (range 3–0.7), and the final 12-month mean BCVA was 1.23 ± 0.56 logMAR (20/400 Snellen; range 3–0.5 logMAR; 20/20,000–20/63 Snellen; *p* < 0.05) (Table 1).

**Figure 1:**
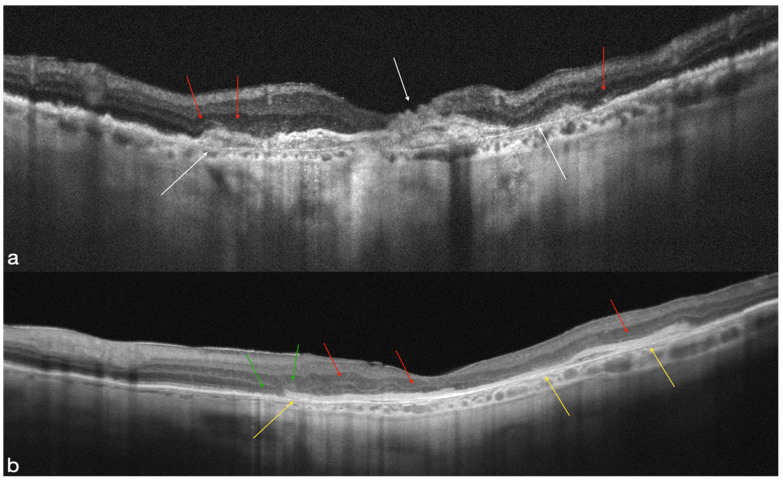
The retinal layers are disrupted atrophically in preoperative OCT (**a**) (white arrows). Evidence of ELM and EZ disruption over the atrophy area (red arrows). The hAM patch is well-positioned and supporting the posterior retinal layers, according to postoperative OCT (yellow arrows). The ELM are visible throughout the area of the retina that overlaps the AM (red arrows). There is a presence of an elliptical zone on the nasal margin of the hAM patch (green arrows) (**b**).

**Figure 2:**
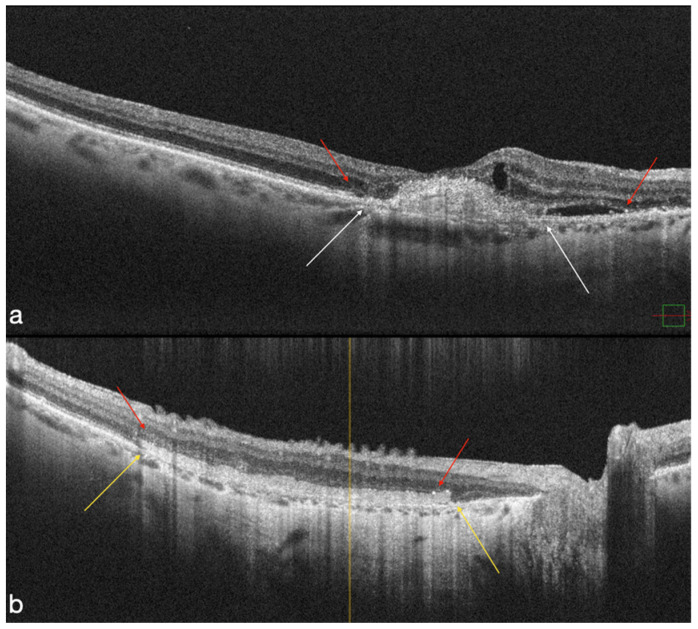
The atrophic breakdown of the retinal layers in the MNV area is visible on preoperative OCT (white arrows). Evidence of ELM and EZ disruption over the atrophy area (red arrows) (**a**). In the macula region, the hAM patch is clearly visible on postoperative OCT (yellow arrows). The ELM are visible throughout the area of the retina that overlaps the AM (red arrows) (**b**).

All patients saw an increase in BCVA of more than two Snellen lines; however, only four patients (18.1%) experienced any alterations to their BCVA, and none of them showed a decline (Figure 1 and Figure 2).

### 4.2. Atrophic Group

The mean BCVA in the five patients with atrophic eyes improved from 1.84 logMAR (20/2000 Snellen), with a range of 20/2000 to 20/800 (2–1.6 logMAR), to 1.26 logMAR (20/400 Snellen), with a range of 20/2000 to 20/100 (2–0.7 logMAR), with a *p*-value of 0.0084. Four patients gained more than two Snellen lines, and one patient did not improve. The average BCVA at 1-, 3-, and 6-month follow-ups was 1.6 logMAR (20/800 Snellen), with a range of 2 to 1 logMAR, 1.38 logMAR (20/500 Snellen), with a range of 2 to 1 logMAR, and 1.28 logMAR (20/400 Snellen), with a range of 2 to 0.8 logMAR, respectively (Table 2).

### 4.3. OCT-A Results

**Figure 3:**
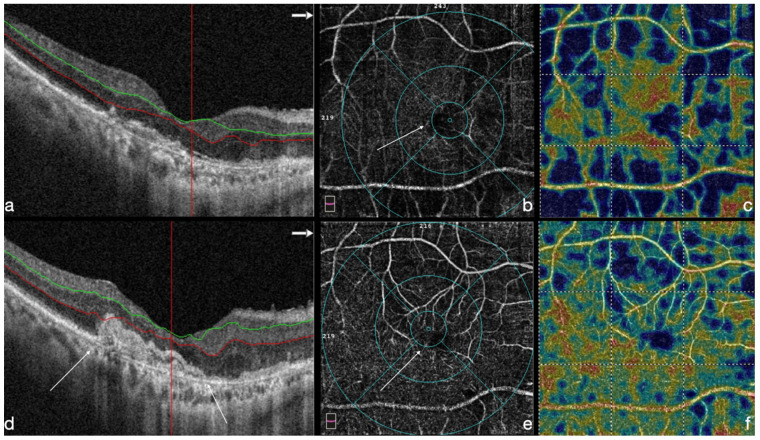
Preoperative structural OCT (**a**) and OCT-A deep vascular density (DVD) in advanced AMD (**b**,**c**). Values for foveal, parafoveal, and total DVD are 21.3%, 47.0%, and 34.5%. The angio scans show an absence of the deep capillary plexus in macular area with an enlargement of the deep foveal avascular zone (FAZ) (white arrows) (**b**,**c**). Six months postoperative structural OCT shows the presence of the hAM (white arrows) (**d**). A-OCT DVD (**e**,**f**) shows values for foveal parafoveal and total of 32.9%, 47.9%, and 45.7%, respectively, with a reorganization of the deep capillary plexus and the FAZ area (white arrow). Green and red lines (**a**,**d**) indicates the boundaries of the inner nuclear layer.

OCT-A analysis focused on deep vascular density (DVD) variations over the follow-up period in the entire cohort. Preoperative values for foveal, parafoveal, and total DVD were 21.6 ± 4.8%, 47.0 ± 6.2%, and 34.5 ± 5.1%. At the end of the follow-up period, a significant increase in parafoveal (51.2 ± 7.2%) and total (38.0 ± 6.8%) DVD were reported (*p* = 0.02 and *p* = 0.03, respectively). OCT-A vascular density was correlated with the final 12-month BCVA, and a high correlation resulted for the total and parafoveal deep vascular density (*r* = −0.48 and −0.63, respectively, *p* < 0.001). FAZ analysis showed no correlation with the final BCVA (Figure 3). Finally, no significant differences were found in OCT-A parameters between MNV and GA-groups (*p* = 0.38).

## 5. Conclusions

Our method utilizing the hAM to treat patients with severe AMD, showed good anatomical outcomes, a reasonably simple surgical technique, outcomes that are comparable to those of more entangled procedures, good visual acuity recovery, inhibition of MNV recurrence, and a decrease in anti-VEGF postoperative injections. This treatment has shown to be safe and to have no visible immunologic reactions.

The hAM is a pluripotent tissue, and while it might not be enough to significantly repair an advanced AMD disease, we suggest that using it in combination with other regenerating therapies may produce better results and a satisfying recovery of visual acuity. These early results need to be confirmed with novel research, with bigger cohorts and longer follow-ups, in order to clarify whether the hAM acts as a regenerative stimulus or as a scaffold for photoreceptorial migration.

## Figures and Tables

**Table 1:** Demographic and ophthalmologic preoperative and postoperative characteristics in the MNV group.

Patient ID/Age (YO)/Sex	Eye	Lens Status	Preoperative BCVA—Snellen (LogMAR)	1-Month BCVA—Snellen (LogMAR)	3-Month BCVA—Snellen (LogMAR)	6-Month BCVA—Snellen (LogMAR)	12-Month BCVA—Snellen (LogMAR)
1/70/F	Right	phakic	20/2000 (2)	20/400 (1.3)	20/100 (0.7)	20/2000 (2)	20/400 (1.3)
2/68/M	Right	pseudophakic	20/2000 (2)	20/2000 (2)	20/400 (1.3)	20/400 (1.3)	20/200 (1)
3/80/F	Right	pseudophakic	20/2000 (2)	20/2000 (2)	20/400 (1.3)	20/100 (0.7)	20/100 (0.7)
4/80/M	Right	phakic	20/2000 (2)	20/2000 (2)	20/2000 (2)	20/2000 (2)	20/2000 (2)
5/72/F	Left	pseudophakic	20/2000 (2)	20/2000 (2)	20/2000 (2)	20/400 (1.3)	20/400 (1.3)
6/68/M	Right	phakic	20/2000 (2)	20/400 (1.3)	20/400 (1.3)	20/400 (1.3)	20/400 (1.3)
7/85/F	Left	pseudophakic	20/2000 (2)	20/2000 (2)	20/800 (1.6)	20/800 (1.6)	20/400 (1.3)
8/86/F	Left	pseudophakic	20/2000 (2)	20/2000 (2)	20/400 (1.3)	20/400 (1.3)	20/400 (1.3)
9/79/M	Right	pseudophakic	20/800 (1.6)	20/200 (1)	20/200 (1)	20/125 (0.8)	20/100 (0.7)
10/85/M	Right	pseudophakic	20/800 (1.6)	20/200 (1)	20/200 (1)	20/200 (1)	20/200 (1)
11/87/M	Right	pseudophakic	20/2000 (2)	20/2000 (2)	20/2000 (2)	20/400 (1.3)	20/200 (1)
12/83/F	Left	phakic	20/2000 (2)	20/2000 (2)	20/2000 (2)	20/2000 (2)	20/2000 (2)
13/75/F	Left	phakic	20/200 (1)	20/200 (1)	20/160 (0.9)	20/100 (0.7)	20/80 (0.6)
14/84/M	Right	pseudophakic	20/2000 (2)	20/400 (1.3)	20/400 (1.3)	20/200 (1)	20/200 (1)
15/73/M	Left	phakic	20/2000 (2)	20/160 (0.9)	20/160 (0.9)	20/200 (1)	20/125 (0.8)
15/73/M	Right	phakic	20/20,000 (3)	20/400 (1.3)	20/400 (1.3)	20/400 (1.3)	20/400 (1.3)
16/80/F	Right	pseudophakic	20/2000 (2)	20/2000 (2)	20/2000 (2)	20/2000 (2)	20/2000 (2)
17/88/F	Left	pseudophakic	20/2000 (2)	20/2000 (2)	20/400 (1.3)	20/400 (1.3)	20/400 (1.3)
18/80/M	Right	pseudophakic	20/800 (1.6)	20/2000 (2)	20/400 (1.3)	20/400 (1.3)	20/200 (1)
19/78/M	Right	pseudophakic	20/800 (1.6)	20/200 (1)	20/200 (1)	20/200 (1)	20/200 (1)
20/78/F	Right	phakic	20/20,000 (3)	20/20,000 (3)	20/20,000 (3)	20/20,000 (3)	20/20,000 (3)
21/75/F	Left	pseudophakic	20/400 (1.3)	20/200 (1)	20/200 (1)	20/100 (0.7)	20/80 (0.6)
21/75/F	Right	pseudophakic	20/200 (1)	20/200 (1)	20/125 (0.8)	20/100 (0.7)	20/63 (0.5)

YO: years old; BCVA: best corrected visual acuity.

**Table 2:** Demographic and ophthalmologic preoperative and postoperative characteristics in the geographic atrophy group.

Patient ID/Age (YO)/Sex	Eye	Lens Status	Preoperative BCVA—Snellen (LogMAR)	1-Month BCVA—Snellen (LogMAR)	3-Month BCVA—Snellen (LogMAR)	6-Month BCVA—Snellen (LogMAR)	12-Month BCVA—Snellen (LogMAR)
1/80/M	Right	phakic	20/2000 (2)	20/2000 (2)	20/2000 (2)	20/2000 (2)	20/2000 (2)
2/85/F	Left	pseudophakic	20/2000 (2)	20/2000 (2)	20/800 (1.6)	20/400 (1.3)	20/400 (1.3)
3/86/F	Left	pseudophakic	20/2000 (2)	20/2000 (2)	20/400 (1.3)	20/400 (1.3)	20/400 (1.3)
4/79/M	Right	pseudophakic	20/800 (1.6)	20/200 (1)	20/200 (1)	20/125 (0.8)	20/100 (0.7)
5/85/M	Right	pseudophakic	20/800 (1.6)	20/200 (1)	20/200 (1)	20/200 (1)	20/200 (1)

YO: years old; BCVA: best corrected visual acuity.

## Data Availability

Data are contained within the article or Supplementary Materials.

## Supplementary Materials

The following supporting information can be downloaded at: https://www.mdpi.com/article/10.3390/life12121998/s1, Video S1: Surgical Technique.
